# Know Thy Neighbor: Costly Information Can Hurt Cooperation in Dynamic Networks

**DOI:** 10.1371/journal.pone.0110788

**Published:** 2014-10-30

**Authors:** Alberto Antonioni, Maria Paula Cacault, Rafael Lalive, Marco Tomassini

**Affiliations:** Faculty of Business and Economics, University of Lausanne, Lausanne, Switzerland; Center of Nonlinear Studies, China

## Abstract

People need to rely on cooperation with other individuals in many aspects of everyday life, such as teamwork and economic exchange in anonymous markets. We study whether and how the ability to make or break links in social networks fosters cooperate, paying particular attention to whether information on an individual's actions is freely available to potential partners. Studying the role of information is relevant as information on other people's actions is often not available for free: a recruiting firm may need to call a job candidate's references, a bank may need to find out about the credit history of a new client, etc. We find that people cooperate almost fully when information on their actions is freely available to their potential partners. Cooperation is less likely, however, if people have to pay about half of what they gain from cooperating with a cooperator. Cooperation declines even further if people have to pay a cost that is almost equivalent to the gain from cooperating with a cooperator. Thus, costly information on potential neighbors' actions can undermine the incentive to cooperate in fluid networks.

## Introduction

Cooperation is a widespread behavior and a necessary condition for the advancement of social institutions and society as a whole. However, cooperation may easily fall prey to exploitation by selfish individuals who only care about short-term gain [1]. For cooperation to evolve, specific conditions and mechanisms are required, such as kinship, direct and indirect reciprocity through repeated interactions, or a particular structure in the interaction networks that are the fabric of society (see, e.g., [2] for a concise and insightful summary of a vast amount of work, or [3] for a presentation in layman's terms). This network reciprocity does not require any particular psychological propensity or behavior on the part of the agents, but only a heterogenous distribution of the individuals in the interacting populations. Both theory and quantitative simulations indicate that network reciprocity explains the implicit cooperation that is at the core of society (see, e.g., [4]–[6]). To summarize these results, the mere presence of a spatial or relational structure gives rise to evolutionary outcomes in which, thanks to positive assortment, cooperative behavior may evolve and may even lead to fully cooperative states. Recent research tested these predictions by means of targeted experiments with humans in the laboratory, in which the subjects were connected in specific network structures [7]–[10]. Surprisingly, these studies found that neither homogeneous nor heterogeneous network structures promote cooperation to a significant extent [8], [9], [11].

The above analyses relate to networks that do not change with time. However, many actual socio-economic networks are dynamic. This fact has not escaped the attention of researchers, and several models have been proposed for studying cooperation under these conditions (see, e.g., [12]–[21] among others, and the recent review in [22]). There are also model studies of the coevolving ultimatum game [23].

These models differ in their details, but researchers agree that adding these new adjustment margins may lead populations to mainly cooperative and stable states through co-evolution of behavior and connectivity. Empirical tests of dynamic settings include [24]–[27]. Rand et al. [24] found that cooperation is supported if participants can rewire connections often enough, and that the evolved networks are more heterogeneous and have more stable links between two cooperators than in less fluid or completely static conditions. Wang et al. [26] investigated the role of link updating frequency on cooperation and found that partner updating significantly increased the cooperation level even at relatively low frequencies.

We study a setting where individuals can make or break links and need to pay for information on their potential partners' actions. This setting differs from Rand et al. [24] who provided the players with full information on the strategies used by their neighbors in the previous round. Also, Wang et al. [26] provided even richer information, again for free. Players were shown the identities (anonymous labels) and action choices of all players with whom they were connected for up to five previous rounds. While we think that these conditions could be adequate in some situations in which the same people interact repeatedly, we argue that there are many contexts in which there is uncertainty as to potential partners' actions and, by consequence, a decision entails some amount of risk. This is the case in today's widespread large online systems, where changing and multiple identities are easy to create and use and confer a certain amount of anonymity to the participants. We believe that this is an important situation to investigate, and we have tried to introduce this factor in our experiments by imposing a cost on a player to “discover” the current strategy of a potential partner.

## Experimental Setup

Our experimental setup is based on the Prisoner's Dilemma game [28]–[30]. In this two-person game, players must decide whether to cooperate or to defect. If both cooperate, each gets a payoff 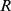. If one defects and the other cooperates, the defector gets 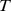 and the cooperator receives the payoff 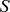. If both defect, each gets 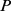. Since 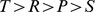, defection is a dominating strategy and a rational payoff-maximizing player will choose to defect, although mutual cooperation yields a higher collective payoff, whence the dilemma. Evolutionary reasoning leads to the same result, as defectors will reproduce at a higher rate due to their superior payoff [31]. This simple game perfectly displays the tension between socially desirable outcomes and self-interested individual actions. In our experiment, subjects played a Prisoner's Dilemma game with their immediate neighbors in the network, with 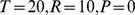, and 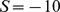. These payoff values are the same as those used in [24], except for an uninfluential scale factor. The initial set of connections between the participants was chosen to be a regular random graph of degree 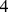. Participants played 15 periods of the game described below, although this exact number was unknown to them; they were only told that they would play for at least 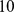 periods. Each period consisted of the following five stages:

1. Action choice

2. Link proposals

3. Information acquisition choice

4. Link acceptance decision

5. Feedback on payoffs

In the first stage, players had to select one of two actions, “square” or “circle,” where “square” implied “cooperation” and “circle” implied “defection.” We chose to label actions in a neutral fashion to rule out framing effects. The association between the label (“circle” or “square”) and the actions (cooperation or defection) was randomized across sessions.

In the second stage, subjects received information on their own action and the number of current neighbors that selected each of the two actions. Subjects then chose one and only one of the following actions: do nothing at this stage, break a link with one of their current neighbors who chose “square,” break a link with one of their current neighbors who chose “circle,” or ask to be matched with a randomly chosen individual who is not yet their neighbor.

In the third stage, subjects saw how many individuals wanted to link with them (those who asked to be linked and were randomly assigned to the subject and his or her new partner if he or she asked for one). Subjects decided whether to pay a cost of 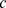 per connection to be informed about the current action (“square” or “circle”) of each potential partner.

In the fourth stage, subjects saw the information they paid for and decided whether or not to accept each pending connection. Link deletion was unilateral. Link creation required mutual consent from both partners.

After these decision stages, subjects were informed of their current payoff as well as their accumulated payoff. They were neither informed about their neighbors' payoffs nor about their neighbors' individual strategy choices; they only knew the number of people playing each of the two strategies among the neighbors. Participants never knew the full network topology.

The crucial experimental variation concerned the cost of obtaining information on the partner's decision in stage three. We implemented a baseline condition with cost 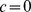. Results from that baseline allow us to compare our design with the existing designs. We expected to obtain similar levels of cooperation, since we implemented a fluid dynamic network design with free information. Crucially, we also implemented two settings in which participants needed to pay before they got access to information on their potential partners' actions. Participants paid a cost of 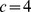 in the low-cost condition and a cost of 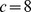 in the high-cost condition. Note that these costs are small in the sense that the cost of getting the information is smaller than the benefit of playing the game with a cooperator, i.e., 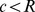. Paying the cost is also rational for a cooperator who fears being paired with a defector, since the cooperator pays 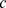 to avoid a payoff of 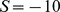 (see file S1 for details on rational behavior analysis).

We repeated this experiment twice for each group of 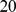 participants. We re-initialized the network to be a new regular random graph of degree 4, and participants played the same game in the same treatment condition for another 15 periods.

## Results

**Figure 1:**
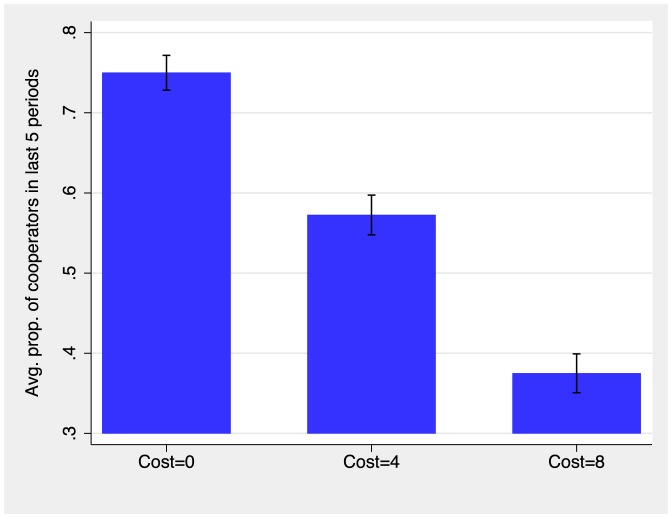
Fraction of individuals cooperating during the last 5 periods by cost of obtaining information on new partner. Cooperation is the dominant action if information on the new partner's action is available for free. Cooperation plummets as costs of obtaining information on new partner's actions are introduced. The capped spikes plot the standard errors of the mean.

We begin by examining the amount of cooperation that prevailed during the last five rounds of the experiment. Figure 1 depicts the average level of cooperation in the last five periods as a function of the information-gathering cost. Recall that participants knew that the experiment would last at least 10 periods. During periods 11 through 15, participants expected that the experiment could end in any period. This feature could have triggered “last round” effects, where participants typically return to the Nash Equilibrium of the stage game. The final five rounds are therefore a strong test for cooperation.

Results in Figure 1 indicate that cooperation attains a high level when information is freely available (Cost = 0). About three out of four decisions are cooperation decisions (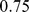). Cooperation declines substantially once information on the potential partner's action is introduced. Merely two out of five players cooperate in the high-cost treatment (the fraction cooperating is 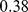, significantly lower than in the no-cost treatment, 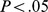). Cooperation levels are also lower if participants need to pay an intermediate cost in order to access information on their future partners' actions (the fraction cooperating is 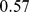, significantly lower than in the no-cost treatment, 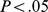). Thus, high costs for information on potential partners' actions hurts cooperation.

**Figure 2:**
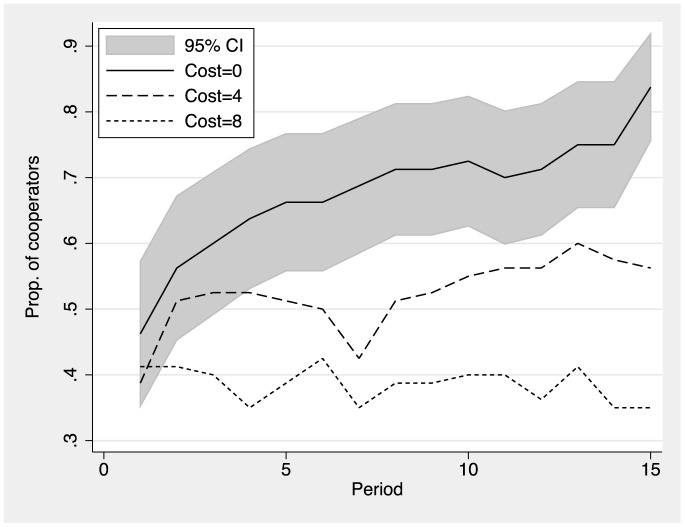
Fraction of cooperators by period and treatment (cost of information). Cooperation starts at just below 50% in all treatments. Cooperation rapidly increases over periods in the treatment with free information on potential partners' actions. Cooperation builds up less rapidly in the low-cost (Cost = 4) treatment and remains almost at the initial level for the high cost (Cost = 8) treatment. The grey area plots the 95% CI for the Cost = 0 treatment.

We now turn to the evolution of cooperation over time. Fig. 2 reports the fraction of cooperators per period for the three different cost treatments. The shaded area is the 95% confidence interval for the baseline treatment (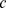 = 0). Cooperation increases steadily when information is free. About 80% of all participants decide to cooperate in the final period. Cooperation also increases in the low-cost treatment with 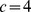, but merely 55% of all subjects cooperate in the final round. Cooperation ceases to build up over time in the high-cost treatment with 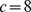. Merely 35% of all individuals cooperate in the final period. Thus, the cost of obtaining information on a future partner's action dramatically reduces the capacity of fluid networks to sustain cooperation.

**Figure 3:**
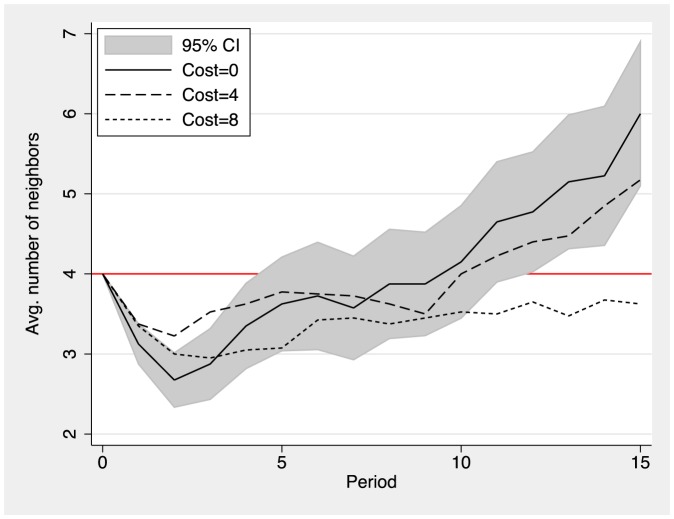
Average number of neighbors by period and treatment (cost of information). The grey area plots the 95% CI for the cost = 0 treatment.

**Figure 4:**
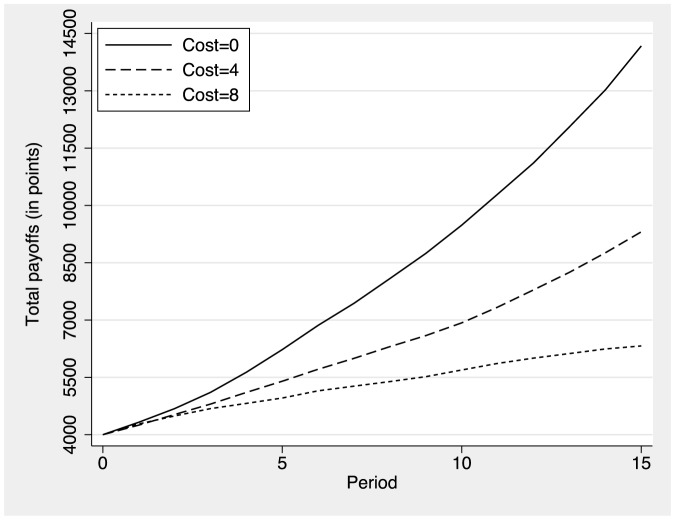
Total wealth in the population by period as a function of the cost required to discover information on partners. Participants start with an initial endowment of 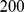 points each.

In the following we discuss the effects of costly information on the average number of neighbors of each participant, i.e., the participant's degree. Figure 3 shows the average number of neighbors by period for the different cost treatments. Initially, all individuals have four neighbors in all three conditions. Interestingly, subjects initially sever a number of existing links and average degree decreases from the initial level of four neighbors to a level of just under three neighbors between period 0 and period 3 (see file S1 for a detailed analysis of link proposal decisions). Average degree then increases rapidly and reaches a level of about 6 links in the free-information condition. Average degree increases to a very similar extent in the low-cost condition, with participants having about five neighbors in the final round. Participants who had to pay the high cost also accepted new links but at a much lower rate. Average degree remains below the initial level of four neighbors in the high cost condition. This analysis indicates that the social networks become sparser as the cost of obtaining information on neighbors' actions increases. Related results that tend to confirm this trends by numerical simulations have been published by J. Tanimoto [32], [33]. The total accumulated payoff earned by all members of the population is clearly related to the number of neighbors and the fraction of cooperators. This quantity is shown in Fig. 4 where it is clearly seen that a higher mean degree in a population mainly composed by cooperators is highly favorable for social wealth.

**Figure 5:**
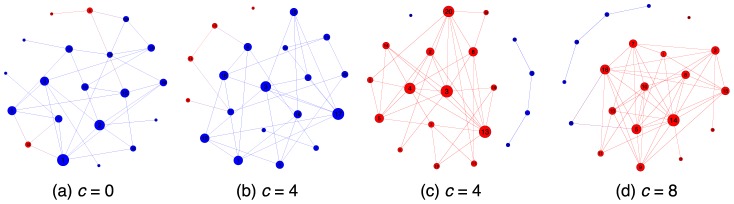
This figure displays the final state of four networks. Blue stands for cooperation and red for defection. The size of each node represents the number of neighbors of a node. The figure shows that the cost of obtaining information dramatically alters the final state of the network. The free information treatment results in a majority of cooperators playing each other in a densely connected social network (a). The high-cost treatment results in a majority of defectors, again fairly densely connected (d). The low-cost treatment produces a case that is similar to the low-cost treatment (compare (b) to (a)) or a state that is similar to the high cost treatment (compare (c) to (d)). Cooperation in dynamic social networks exhibits path dependence in the low-cost treatment.

**Figure 6:**
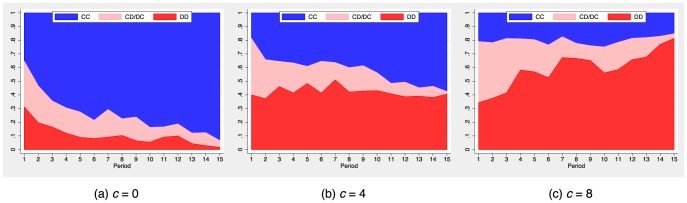
Evolution of link type by cost of obtaining information. Links between two cooperators are shaded blue, links between two defectors are shaded solid red, and mixed links are shaded light red. Links between two cooperators displace other link types almost completely in the free information treatment (a). Links among two defectors displace the other link types almost completely in the high-cost treatment (c). The low-cost treatment has either cooperator or defector links, depending on the final state of the network (b).

We now turn to how the cost of obtaining information on neighbors affects the resulting social networks. Fig. 5 displays the topology of networks that formed in the final period. Fig. 5 presents the final topology we obtained in four of the total 12 runs of the experiment. These four topologies are representative of the total we obtained (see Figs. 4, 5, and 6 in the file S1 for all final topologies). Network (a) displays the final network state in cases in which information on neighbors' actions is freely available. This network consists of densely connected cooperators with only a few poorly connected defectors scattered around. Networks (b) and (c) resulted from the low-cost (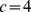) treatment. They are representative of the two tendencies we observed for this cost value. Players either tended toward a clear majority of cooperators, as in (b) (very similar to 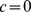 in case (a)), or they tended towards a state with many defectors and only a few poorly connected cooperators (see image (c)). Network (d) resulted from the treatment with 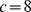 and is typical of this condition: defectors prevailed in all cases. Interestingly, it appears that the cost 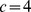 is a threshold cost such that for cost values less than 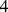 the population tends to self-organize in a mainly cooperative structure, while for higher costs people seem to be more conservative and tend toward defection. Information-gathering costs are important for the emergence of cooperation in dynamic anonymous networks.

We now turn to a more detailed discussion of how players interacted with their neighbors. Fig. 6 shows the frequency of links that involved two cooperators (CC), two defectors (DD), or one cooperator and one defector (CD/DC). The population ends up mostly cooperating, and the majority of links are between cooperators in the baseline treatment (see case (a)). Links initially involve two cooperators only in 35% of all cases, but their frequency steadily increases and displaces both links among defectors and the mixed links. Introducing a low cost of obtaining information on neighbors' actions dramatically changes the dynamics of link types. Both links involving only cooperators and links involving only defectors can be present in the final state, depending on the type of final network (see case (b)). The picture fundamentally changes with high costs. Links involving two defectors now displace mixed links. Cooperator links remain fairly stable but at a low level. Interestingly, 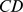 links tend to disappear, meaning that subjects refuse to be exploited and punish defectors by severing links to them. Note also that links between two defectors are stable in our setting because participants neither gain nor loose in that interaction.

**Figure 7:**
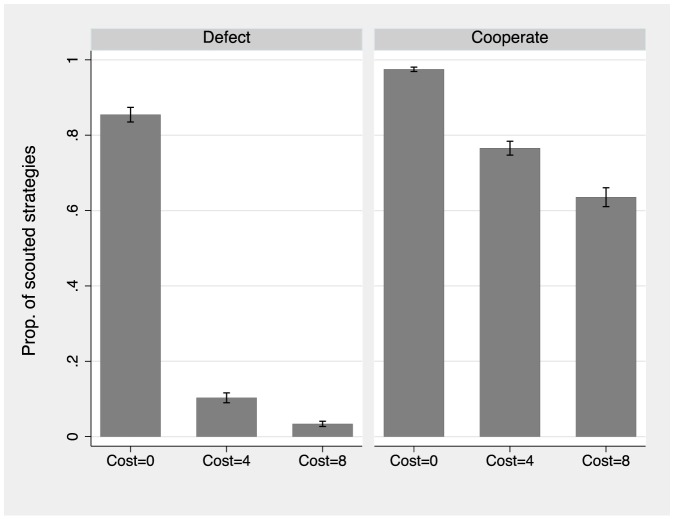
Fraction of scouted links among potential links by individual action and treatment (cost of information). “Scouting” means that the player asked to see the action of the potential neighbor. Both defectors and cooperators scout when information is freely available. Defectors almost cease to scout once information on the neighbor's action becomes costly. Cooperators continue to scout as information becomes costly, albeit at a lower rate than in the free-information treatment. The capped spikes plot the standard errors of the mean.

Now we discuss how the cost of obtaining information on neighbors' actions affected the demand for that information. Figure 7 presents information on how many subjects wanted to know the strategy of a potential new partner; we call this action “scouting.” The figure plots the proportion of scouted strategies as a function of the information cost, for both cooperators and defectors. The majority of participants chose to discover the decision of a potential partner when it was free, regardless of their own decision. However, their behavior was different when scouting entailed a cost. Defectors were reluctant to pay for the information on their neighbors' action because they did not incur losses when playing another defector (both earned the payoff 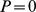), and they gained by playing a cooperator (defector gains 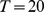). In contrast, cooperators incurred losses when playing a defector, so knowing the potential partner's strategy continued to be valuable to them. Nonetheless, the fraction of cooperators paying to scout their future neighbors' action decreased substantially, from 95% in the free-information treatment to below 80% in the low-cost treatment, and to about 60% in the high-cost treatment. This behavior in turn reduced the incentive for players to cooperate, since defection could go undetected in the high-cost treatment.

**Figure 8:**
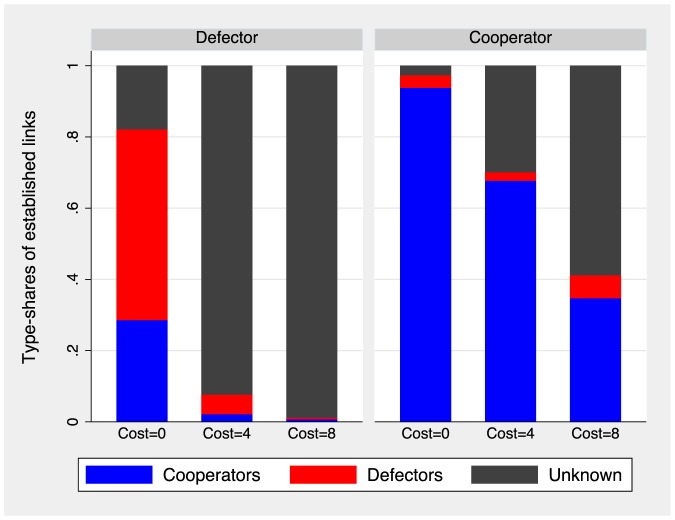
Proportion of established links with defectors, cooperators, and unknown partners according to players' current type and as a function of the cost 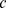.

Finally, we now discuss how costly information shapes link acceptance. Figure 8 plots the proportion of each type of effectively created link by treatment and according to the participants' current strategies. When information is free, defectors create few links with cooperators because the latter do not give their consent. Most new links of defectors are to other defectors, and the rest are to unknown types. Cooperators, on the other hand, always establish links to cooperators and reject defectors except for some rare cases. When information about others' strategies entails a cost, defectors no longer scout and link only to unknown types, with few exceptions. Cooperators continue to pay for the information, since the expected gain is positive (see file S1, rational behavior analysis section). However, the expected gain from acquiring the information decreases with its cost. Hence, the proportion of new links to unknown types increases as the cost rises.

## Discussion

We have experimentally investigated the cooperation behavior in networks where participants playing a Prisoner's Dilemma game are allowed to update their connections by making and breaking ties. While recent experiments have focused on contexts in which information on the types of players is either fully available or easy to obtain, such as in groups that interact frequently [24], [26], our conditions resemble to today's online networks in which a certain degree of anonymity is easy to achieve. The players only know how many of their current direct neighbors are cooperators and how many are defectors; information on the rest of the network and on the neighbors' payoff is not provided. A subject may suppress an unwanted relationship just by cutting the corresponding link unilaterally at no cost, but forming new links to an unknown participant can be done either “blindly” at no cost or by paying a certain cost to know the participant's current type, i.e., whether he or she is a cooperator or a defector, and the link is effectively created only if both partners agree to accept it. We experimented with two values for the cost of acquiring the information about the potential partner's type: one that is relatively low with respect to the payoff values used in the experiment, and another which is higher but not so high that a rational player would never choose to pay for it. We compared results in the costly setting with a baseline situation in which the information is available for free.

In the baseline free treatment, the network quickly evolved toward full or almost full cooperation. The resulting networks had a high average degree, since it is beneficial for a cooperator to have many links to other cooperators. At intermediate cost an interesting phenomenon emerges, wherein the population may evolve in two ways: either cooperators or defectors may be in the majority. In the high-cost case we are again in a rather clear-cut situation: introducing the high cost favors defection because participants are more reluctant to pay for linking information. The consequence is that cooperators have trouble finding other cooperators to form stable clusters, and the resulting networks are sparser.

Our results suggest that there is a cost barrier that depends on the actual payoffs, beyond which people have less propensity to pay for information, in spite of the fact that knowing the potential partner type improves decision making. We believe that these conditions are representative of many of today's network relationships, in which a certain degree of anonymity can be maintained, and thus our results should be relevant in these contexts. As a consequence, cooperation is more difficult to achieve in situations in which costly or uncertain information about a partner's behavior is the rule. The present work could be extended in a number of directions. For example, in the present setting, breaking a link has no cost and does not require the agreement of the current partner. However, there are situations in which this is not possible, e.g., when there is a contract or when breaking the link would entail adverse social consequences. One could also consider different payoffs for the game, such as having a negative 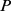, and their influence on cooperation and network dynamics.

## Methods

### Ethics Statement

This research was approved by the ethics committee on the use of human subjects in research of Lausanne University. All participants signed an informed consent describing the nature of the experiment before they entered into the laboratory.

### Procedure

We conducted a total of six experimental sessions in October 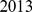. Participants were recruited from the pool of undergraduate students from all disciplines of the University of Lausanne and the Ecole Polytechnique Fédérale of Lausanne using ORSEE [34]. Subject-subject anonymity was granted at all stages, and the experiment was computerized using the z-Tree environment [35]. The use of human subjects in this experiment has been approved by the ethics committee of the University of Lausanne, and participants signed an informed consent describing the nature of the experiment before they entered into the laboratory. Before making decisions, participants read detailed instructions and responded to a set of control questions that insured common understanding of the game and the computation of payoffs. A translation of these instructions from the original French is provided as File S1 to this paper. Each session included 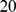 participants (a total of 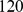 subjects took part in the experiment) and lasted about one and a half hours. Participants received a show-up fee of 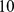 CHF (about $11 USD), and their final score in points was converted at an exchange rate of 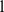 CHF = 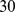 points. The average payoff per student was 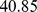 CHF (about $46.2 USD).

All statistical analyses are at the level of the individual using linear regression. Because multiple observations of an individual are not independent, we cluster observations at the individual level. Because individuals play in dynamic networks, we also allow for arbitrary clustering of the error terms between individuals who were neighbors in period 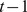. Our results are not sensitive to allowing for clustering at the session level. Differences in cooperation were assessed by means of two dummy variables (Cost 4 takes the value 1 if the individual faced cost 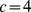, and zero otherwise; Cost 8 takes the value 1 if the individual faced 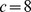, and zero otherwise; see file S1).

## Supporting Information

File S1 **File S1 provides supplementary results on link proposal decisions and final topologies (section 1), presents the statistical analyzes of the main results on cooperation (section 2), discusses whether the scouting decision is rational (section 3), and a translation of the instructions that participants received (section 4).**
(PDF)
