# Supplementary material for: Know Thy Neighbor: Costly Information Can Hurt Cooperation in Dynamic Networks
Source: PLoS One. 2014 Oct 30;9(10):e110788. doi: 10.1371/journal.pone.0110788 (PMC4214703; doi:10.1371/journal.pone.0110788)
Supplement: File S1 — provides supplementary results on link proposal decisions and final topologies (section 1), presents the statistical analyzes of the main results on cooperation (section 2), discusses whether the scouting decision is rational (section 3), and a translation of the instructions that participants received (section 4). (PDF) [file pone.0110788.s001.pdf]

Know Thy Neighbor: Costly Information Can Hurt Cooperation on Dynamic Networks Supplementary Information Appendix

A. Antonioni, M. P. Cacault, R. Lalive, and M. Tomassini

The supplementary information (SI) provides supplementary results on link proposal decisions and final topologies (section 1), presents the statistical analyzes of the main results on cooperation (section 2), discusses whether the scouting decision is rational (section 3), and a translation of the instructions that participants received (section 4).

1 Supplementary Results

1.1 Link Proposal Decisions

Figure 1, 2, and 3 analyze decisions on link proposals (stage 2). Figure 1 reports the fraction of subjects who ask to break a link, with a defector in almost all cases. About half of all the subjects ask to cut a link with a defector in the first period. The fraction breaking rapidly decreases and reaches a level of about 20 % from period 4 to 15. The cost of acquiring information does not affect the decision to break a link substantially.

Figure 2 reports the fraction of subjects who ask to be matched with a new neighbor. The proportion wanting to make a link is initially at around 30%, but increases rapidly to reach a level of between 60 and 80 %. The proportion wanting to establish a new link is somewhat higher in the treatment condition with free information on neighbor's actions than in the high cost of information treatment.

Figure 3 reports the fraction of players who decide to do nothing at the link proposal stage. This proportion remains at 10 %, a very low level, throughout all periods of play. The proportion of idle players is somewhat larger in the high cost treatment, compared to the free information treatment.

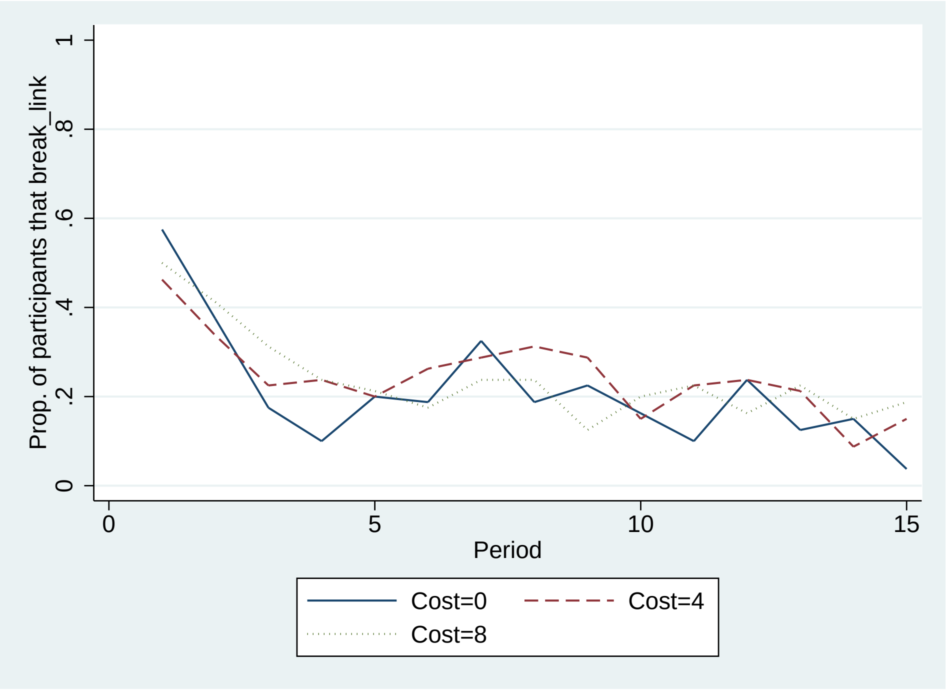

Figure 1: Fraction of players choosing to break a link by period and cost of acquiring information.

1.2 Final Network Topologies

This subsection presents final network topologies in all 12 sessions. We report topoligies

2 Multivariate Regression Analyses

2.1 Linear Regressions

Table 1 presents a statistical analysis of cooperation decisions in the experiment. Recall that all subjects played two repetitions of the experiment. The analysis considers only the decisions of individuals in the first repetition of the experiment to exclude learning effects. The dependent variable in all analyses is a binary variable taking the value 1 if the subject decided to play the cooperative action, and zero otherwise.

It is not trivial to conduct proper inference in networks settings. We have explored three approaches to estimating the standard errors of the parameters measuring the effects of the cost treatment. The first approach reports stan-

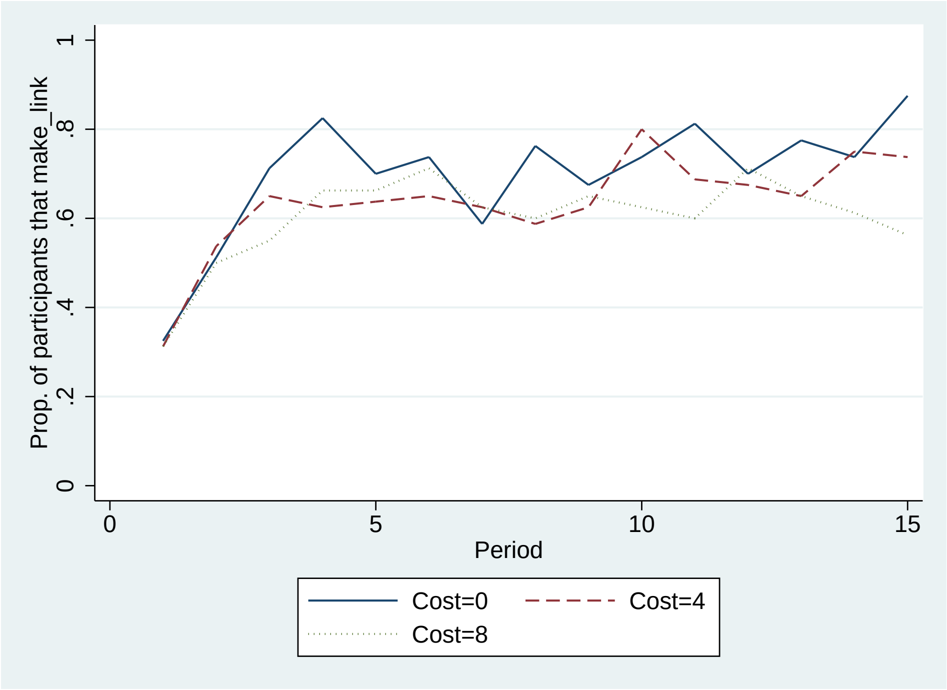

Figure 2: Fraction of players asking to be linked with a new player by period and cost of acquiring information.

dard errors in parentheses in Column (1) of Table 1 that allow for arbitrary clustering across sessions [2]. This approach provides an approximation to standard errors that is appropriate in settings with a large number of clusters. In our setting, the approach is potentially mis-leading since our data is based on six different sessions whereas the clustering approach requires 30 or more clusters.

Our second approach calculates standard errors that allow for arbitrary correlation of errors of individuals that currently are neighbors or have been neighbors at any point in time. The remaining correlations are set to zero. Standard errors from this approach are reported in square brackets in Column (1) of Table 1.

Our third approach to inference applies methods that have been developed in the analysis of spatial data (Column 2 in Table 1). The spatial error model allows for correlation between error terms of different individuals through their linked neighbors [3]. The parameter λ measures the strength of the correlation between error terms of different individuals.

Approaches two and three require that individuals be connected to at least one other neighbor. We discard 92 observations of individuals who did not have any neighbor.

Table 1: OLS, Spatial Error and Logit regressions for cooperation (1st repetition)

	OLS cluster (1)	Spatial Error (2)	Logit (avg. mg. eff.) (3)
Cost4	-0.127	-0.112	-0.123
	(0.054)*	(0.043)***	(0.029)***
	[0.038]**		
Cost8	-0.227	-0.238	-0.222
	(0.078)**	(0.042)***	(0.028)***
	[0.038]***		
Constant	0.583	0.586	
	(0.004)***	(0.030)***	
	[0.027]***		
λ		0.343	
		(0.024)***	
Observations	1588	1588	1588

Notes: (1) Standard errors clustered by session (G-matrix) in parentheses (square-brackets) (2) Standard errors in parentheses (3) Delta-method Standard errors in parentheses. *** P<0.01 ** P<0.05 * P<0.1.

Cooperate=1 if current strategy is cooperate, =0 if defect.

Cost4(8)=1 if participant in cost=4(8) treatment, =0 otherwise.

92 observations are excluded because they were not connected to any other player in the experiment.

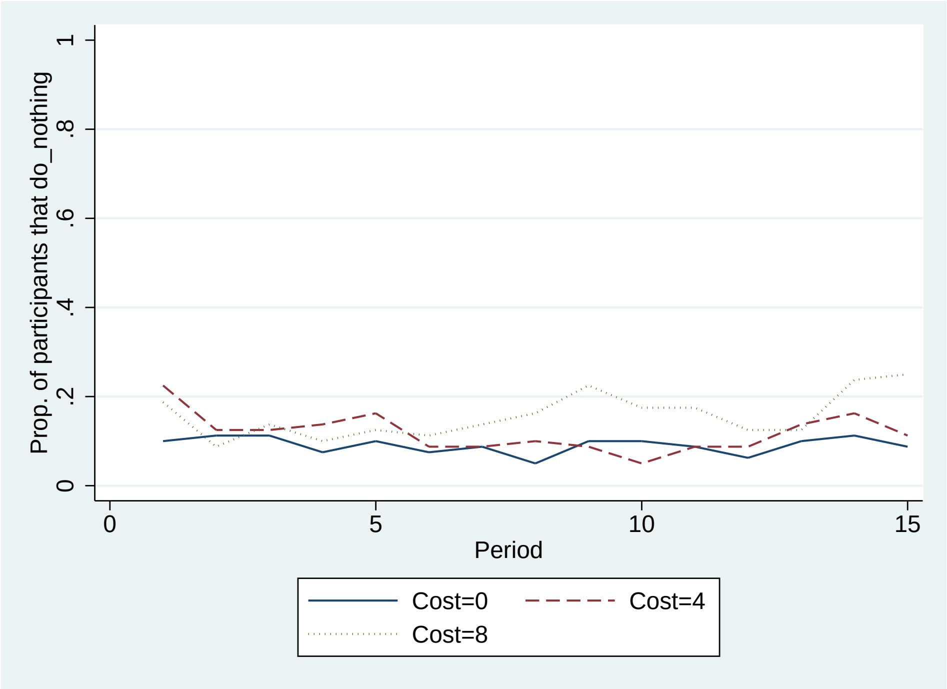

Figure 3: Fraction of players remaining idle by period and cost of acquiring information.

Column (1) of Table 1 presents an ordinary least squares (OLS) analysis of average cooperation decisions across all periods of the experiment. In the free information treatment, 58.3 % of all participants decided to cooperate (see Constant). The cooperation rate was 12.7 % lower in the intermediate cost treatment (Cost4 indicator). The cooperation was 22.7 % lower in the high cost treatment (Cost8 indicator). Column (2) provides somewhat different estimates of the treatment effects since estimates in these columns are based on a maximum likelihood estimation of the spatial error model. The treatment effects are both significantly different from zero, regardless of the approach that we use to assess statistical significance. Our conclusions are robust to the exact approach to assessing statistical significance.

The dependent variable is binary. Column (3) in Table 1 reports estimates of this binary decision variable in a Logit framework. We report the marginal effects of being exposed to an intermediate cost (or a high cost) on cooperation decisions. The treatment effects in the Logit model are virtually identical to effects that are based on OLS analysis (Columns (1) and (2)). Our conclusions are robust to to the exact estimation method chosen to measure the treatment effects. Non-linear Logit analysis and linear OLS methods tend to give similar results in situations where the average of the dependent variable is not too close to either zero or one [1].

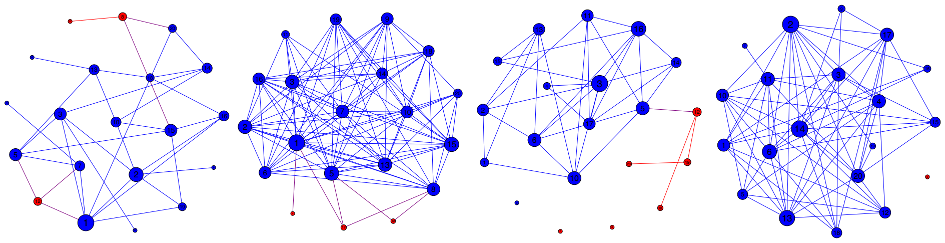

Figure 4: Final network topologies for $c = 0$ treatments. Blue stands for cooperation and red for defection. The size of each node represents the number of neighbors of a node. First and third images represent the sessions performed as first by the subjects, while second and fourth images stand for the second experimental run.

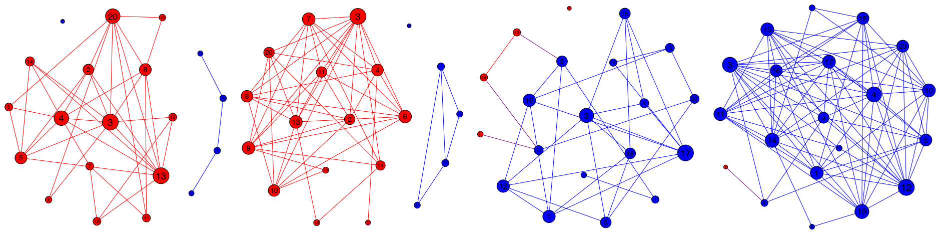

Figure 5: Final network topologies for $c = 4$ treatments.

3 Rational behavior analysis

This section discusses an approach to assessing whether players acted rationally in the model.

3.1 Rational model

Cooperator dynamics

In the rewiring stage cooperators estimate their expected payoffs from the three possible actions: do nothing, cut a link with a defector, and ask for the creation of a new link with a random participant.

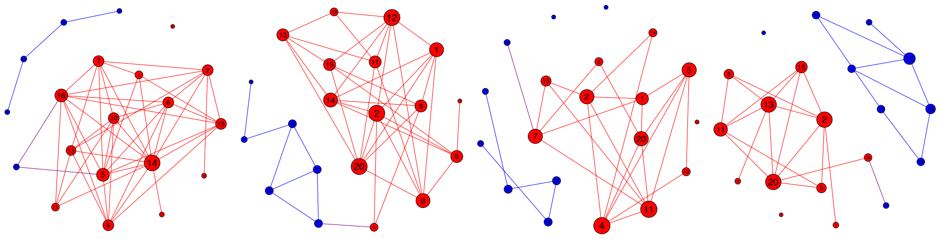

Figure 6: Final network topologies for $c = 8$ treatments.

We define $p_c = p_c(c)$ as the probability that a cooperator pays the cost c in order to know the potential partner strategy. This probability has been estimated empirically as follows:

$p_c \sim 1.0 - 0.05c \rightarrow p_c = 1.0, 0.8, 0.6$ for $c = 0, 4, 8$, respectively.

n_c: current number of cooperators in the current neighborhood
n_d: current number of defectors in the current neighborhood
T, R, P, S: payoffs; $T = 20$, $R = 10$, $P = 0$ and $S = -10$ in our experiment.

Here it follows the estimation of the expected payoffs for a cooperator:

- do nothing:
$$\overline{\Pi}_N = n_c R + n_d S$$
- cut a link with a defector (if any):
$$\overline{\Pi}_C = n_c R + (n_d - 1)S$$
- ask for a new link: this expected payoff is estimated assuming that a cooperator pays the cost c with probability p_c, as explained before. Then, it is assumed that a rational cooperator accepts an unknown potential partner with probability r. This parameter of the model can be seen as the proportion of cooperators in the rest of the population. Figure 7 shows how it is calculated this expected payoff as a function of p_c and r.

  According to Fig. 7:

$$\overline{\Pi}_L = p_c r((n_c + 1)R + n_d S - c) + p_c(1 - r)(n_c R + n_d S - c) +$$

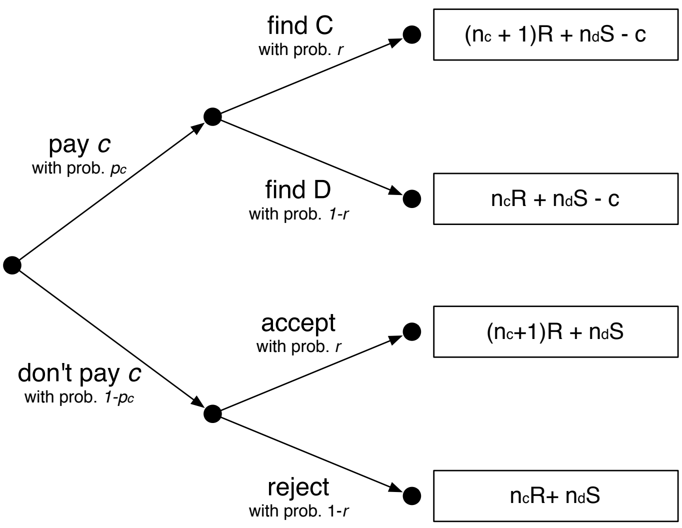

Figure 7: A cooperator pays (or does not) the cost c with probability p_c $(1-p_c)$, while the proposed partner is a cooperator (defector) with probability r $(1-r)$.

$$+(1-p_c)r((n_c+1)R+n_dS)+(1-p_c)(1-r)(n_cR+n_dS)$$

A rational cooperator is thus defined as a cooperator that performs the action which has the best expected payoff among $\overline{\Pi}_N, \overline{\Pi}_C$ and $\overline{\Pi}_L$.

Defectors dynamics

In the rewiring stage a rational defector always ask for a new link.

3.2 Experimental results

Cooperator dynamics. Figure 8 shows the proportion of times a cooperator chooses the *rational* action as a function of r.

Defector dynamics. A defector chose the *rational* action, i.e. ask for a new link, with probability ~ 0.68.

4 Instructions

Each participant read the following set of instructions in detail before the experiment started.

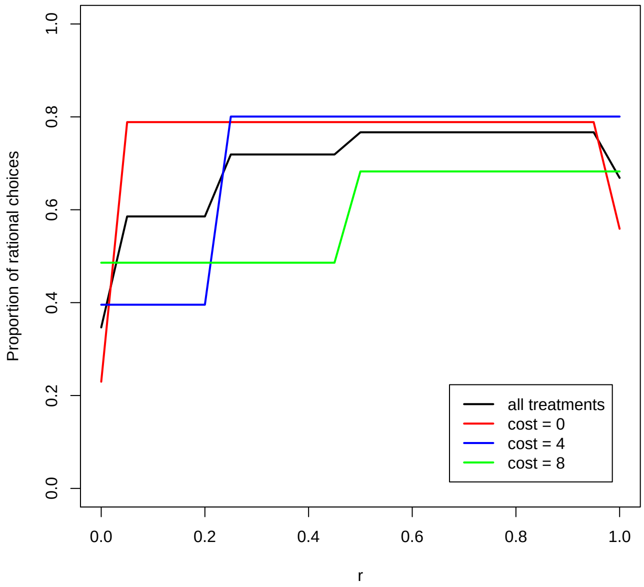

Figure 8:

Explanation for this part of the experiment

(The following instructions were originally written in French.)

Welcome to this experiment!

You will have to make decisions that will affect your income as well as the income of other participants. Although we express all income in terms of points, these points will be exchanged at the end of the experiment using the following exchange rate:

30 pts. = 1.- CHF

From now on, it is **strictly forbidden to talk with other participants**. If you have any questions, please contact the assistants. If you do not follow this rule, we will have to exclude you from the experiment. In this study, each one of the 20 participants interacts with some "neighbors" (other participants in the room) according to a network structure. At the beginning of the experiment all the participants have exactly four neighbors each but this may change during the experiment as you will see below.

During this experiment you will only see your direct neighborhood, i.e. the people that are directly connected to you in the network. You won't be able to see what happens in the rest of the network (for instance, you won't be able to know what the neighbors of your neighbors do).

At the beginning you'll receive a non-renewable endowment of **200 pts.**

What is it about?

There will be at least 10 rounds in the game, but you will not be informed on the exact number. Each round has five stages:

1. Action choice
2. Link proposals
3. Information acquisition choice
4. Link acceptance decision
5. Feedback on payoffs

Those decisions are now explained in more detail below.

1. Action choice

In this first stage you will have to choose an "action" among these two options:

CARRE or CERCLE

Your neighbors will have to make the same decision. Your action is "unique" that is, you'll play this same action against all your direct neighbors (you can't use a different action against different neighbors). Your profit in each round will be calculated as a function of your current action and the strategies chosen by your neighbors.

Here are the gains that you'll get depending on your action and the action of your neighbors:

- you choose CARRE, a neighbor chooses CARRE:
  your payoff is 0 points.
- you choose CARRE, a neighbor chooses CERCLE:
  your payoff is 20 points.
- you choose CERCLE, a neighbor chooses CARRE:
  you loose 10 points (-10 points).
- you choose CERCLE, a neighbor chooses CERCLE:
  your payoff is 10 points.

Your accumulated payoff in each round will be calculated as the sum of the payoffs earned with each one of your current neighbors. The actual neighbors that count for the payoff computation are those with which you'll be linked **at the end of the round** since you will have the possibility of modifying your neighborhood in the next stages, as explained below.

The following examples illustrate your payoff computation at the end of a round. Please note that in the examples you have four neighbors but this number could be different during the experiment. In the drawings you are represented as the central player, whose action is underlined.

Example 1: your action is CERCLE, the action of all your neighbors is also CERCLE.

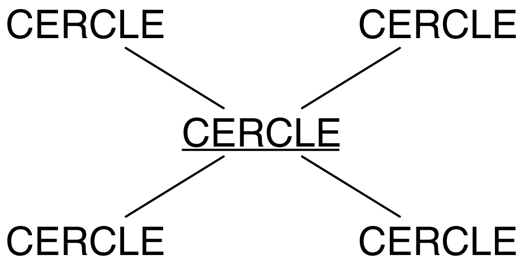

Your payoff is : $10 + 10 + 10 + 10 = 40$ points.

Example 2: your action is CERCLE, the action of all your neighbors is CARRE.

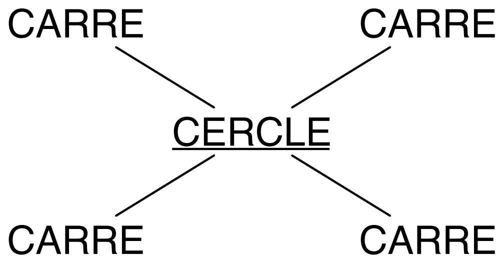

Your payoff is : $-10 - 10 - 10 - 10 = -40$ points.

Example 3: your action is CARRE, the action of all your neighbors is also CARRE.

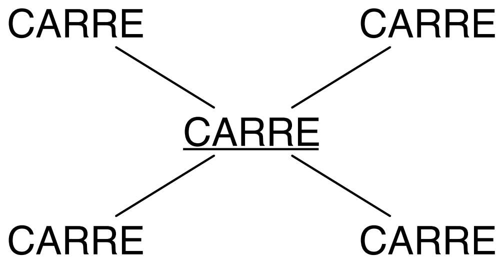

Your payoff is : $0 + 0 + 0 + 0 = 0$ points.

Example 4: your action is CARRE, the action of all your neighbors is CERCLE.

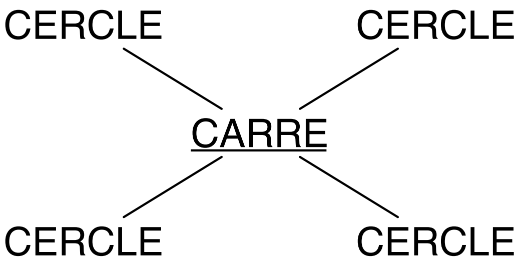

Your payoff is : $20 + 20 + 20 + 20 = 80$ points.

Example 5: your action is CARRE, the action of three of your neighbors is also CARRE while the action of the fourth neighbor is CERCLE.

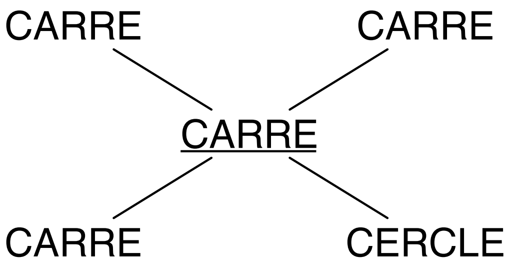

Your payoff is : $0 + 0 + 0 + 20 = 20$ points.

Example 6: your action is CERCLE, the action of two of your neighbors is CARRE, while the action of the two remaining neighbors is CERCLE.

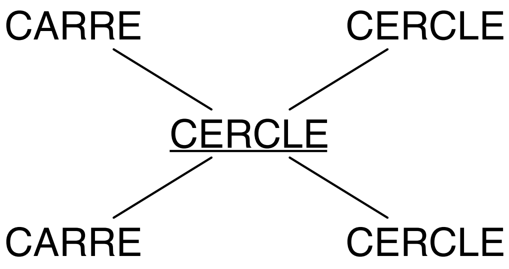

Your payoff is : $10 + 10 - 10 - 10 = 0$ points.

In this stage you will choose the action to adopt in the present round with the help of the following screenshot:

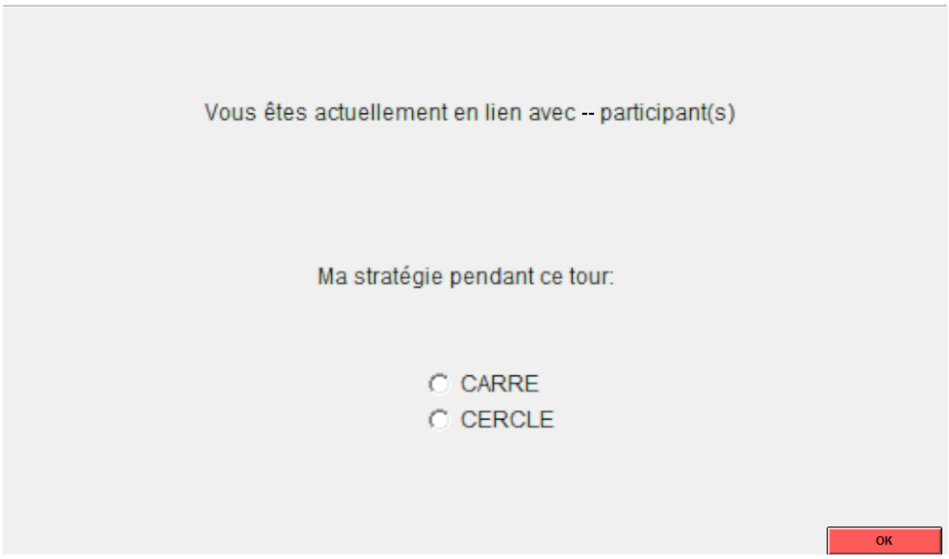

("--" will be replaced by the actual number during the experiment)

2. Link proposals

In this second stage you will be requested to decide whether you would like to change your neighborhood. On the screen you'll see the number of neighbors of each action that you have in this round and you will have to choose one, and only one, of the following actions:

- do nothing in this stage.
- cut a link with a neighbor playing the action CARRE. The link will be deleted automatically.
- cut a link with a neighbor playing the action CERCLE. The link will be deleted automatically.
- create a new link with a randomly chosen participant who is not one of your current neighbors. This link will only be created if both participants accept it in the acceptance stage.

The modifications, if any, will be performed through the following screenshot:

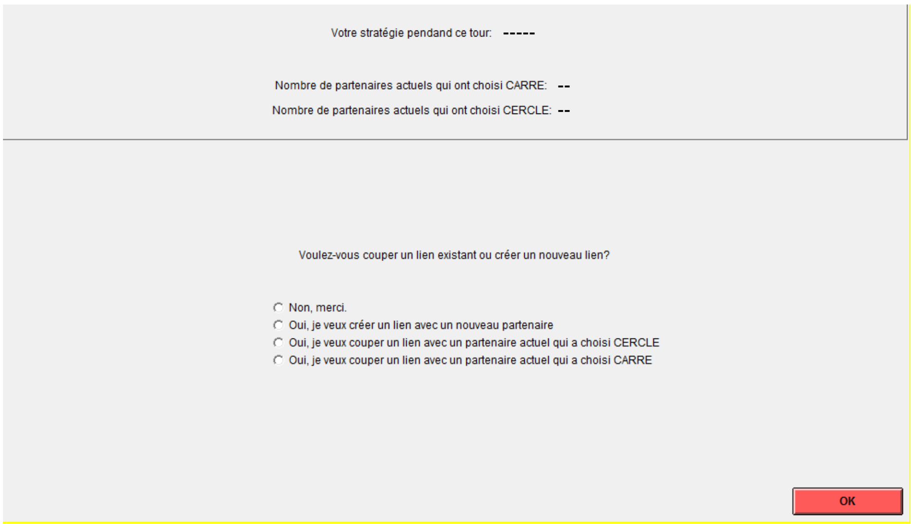

3. Information acquisition

In this stage you may want to know the action of each one of your potential partners. A potential partner is either the random participant that you have asked for, or one or more participants that are asking to create a link to you. The cost for uncovering the action information is **8 points** per potential partner. For example, if you decide to uncover the action of two among your potential partners, you'll pay $2 \times 8 = 16$ points. If you don't have enough points, you won't be able to acquire the information. In this stage you'll be able to implement the previous decision through the following screenshot:

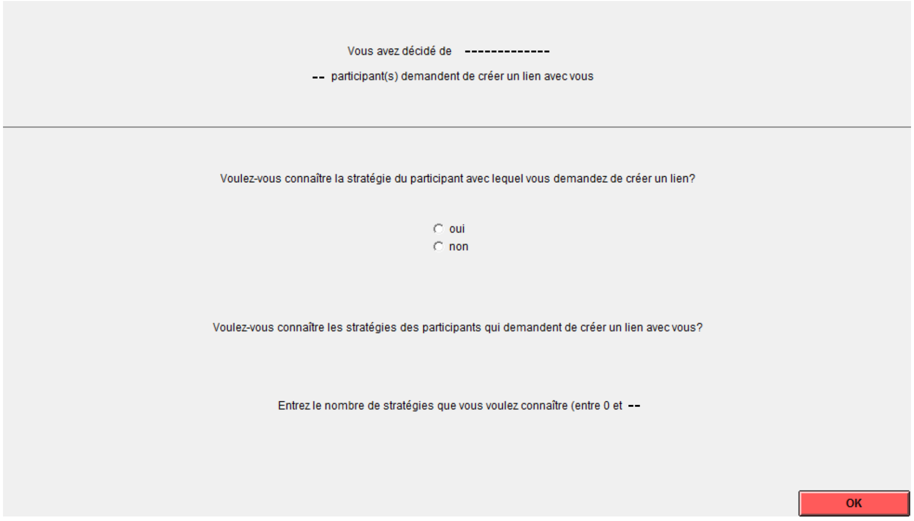

4. Acceptance of new links

Finally, in this stage you are going to decide which links you would like to accept with your potential partners. If you paid to uncover the action of one or more partners in the previous step, those strategies will appear on the screen before you. On the other hand, if you chose not to pay to see them, the corresponding strategies will remain unknown. Please note that a given link will be actually created only if **both partners** have accepted it.

According to the decisions of all participants a new network will be formed. Your payoff for this round, after cost deduction, will be calculated as a function of your action and the action of your current neighbors in the actualized network.

You will complete this stage through the following screenshot:

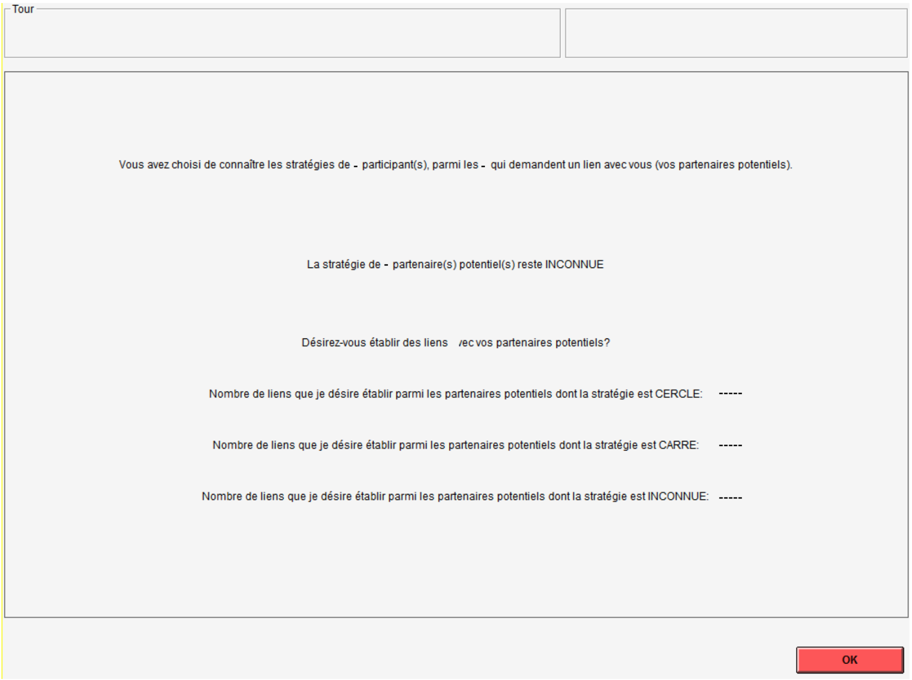

5. Feedback on payoffs

At the end of the current round, a screenshot will appear showing the number of links that have actually been created with you, the number of deleted links to you (note that this could happen even if you didn't ask to cut a link: one or more of your neighbors could have decided to cut the link with you), your current number of neighbors, the respective numbers of the two strategies in

your neighborhood, your payoff for this round, and your total accumulated gain.

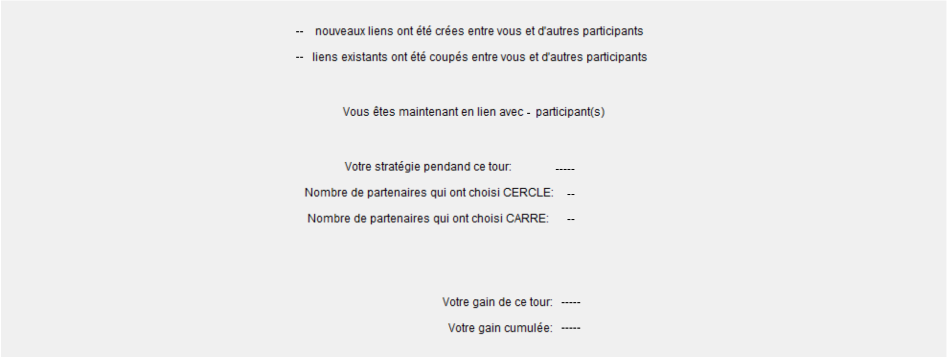

Did you correctly understand the instructions?

Before starting the experiment, we would like to be sure that you and the other participants have correctly understood the decisions that you are going to make. To this end, please answer the questions that will appear on the screen.

References

[1] J. Angrist and S. Pischke. *Mostly Harmless Econometrics: An Empiricist's Companion* Princeton University Press, 2009.

[2] A. C. Cameron and P. K. Trivedi *Microeconometrics: Methods and Applications.* Cambridge University Press, 2005.

[3] A. Clif and K. Ord. Testing for Spatial Autocorrelation Among Regression Residuals. *Geographical Analysis*, 4(3):267-284, 1984.
